# Treatment Outcomes after Dose-Escalated Moderately Hypofractionated Radiotherapy for Frail Patients with High-Grade Glioma

**DOI:** 10.3390/cancers16010064

**Published:** 2023-12-21

**Authors:** Nalee Kim, Hyunju Shin, Do Hoon Lim, Do-Hyun Nam, Jung-Il Lee, Ho Jun Seol, Doo-Sik Kong, Jung Won Choi, Kyuha Chong, Won Jae Lee

**Affiliations:** 1 Department of Radiation Oncology, Samsung Medical Center, Sungkyunkwan University School of Medicine, Seoul 06351, Republic of Korea; nalee.kim@samsung.com (N.K.); hyunjuhaha.shin@samsung.com (H.S.); 2 Department of Neurosurgery, Brain Tumor Center, Samsung Medical Center, Sungkyunkwan University School of Medicine, Seoul 06351, Republic of Korea; dhns.nam@samsung.com (D.-H.N.); jilee.lee@samsung.com (J.-I.L.); hojun.seol@samsung.com (H.J.S.); doosik.kong@samsung.com (D.-S.K.); jungwon8090.choi@samsung.com (J.W.C.); k.chong@samsung.com (K.C.); nswonjae.lee@samsung.com (W.J.L.)

**Keywords:** radiotherapy, brain tumor, hypofractionation, frail patients

## Abstract

**Simple Summary:**

Historically, frail patients who were older and showed poor performance status have been treated with a total dose of 40–45 Gy in 15 fractions, 34 Gy in 10 fractions, or 25 Gy in 5 fractions. We analyzed the oncologic outcomes after dose-escalated fractionated radiotherapy with a total dose of 56 Gy in 20 fractions for high-grade glioma in frail patients who were older than 70 years or showed poor performance status. This dose-escalated regimen with a total dose of 56 Gy in 20 fractions were comparable equivalent dose to conventional radiotherapy with a total dose 60 Gy in 30 fractions for non-frail patients. We observed survival outcomes outperforming historical data. The median overall survival was 12 months. Also, none of these patients experienced severe treatment-related toxicities. Furthermore, salvage treatment (either systemic therapy or local therapy) after progressive disease significantly improved survival outcomes after recurrence compared to supportive care even in frail patients.

**Abstract:**

For high-grade glioma (HGG) patients with old age or poor performance status, hypofractionated radiotherapy (hypoRT) in 10–15 fractions is recommended. Also, limited data exist on the impact of salvage treatment after progression in frail patients. We retrospectively analyzed the outcomes of dose-escalated hypoRT in 40 frail HGG patients who were treated with hypoRT between 2013 and 2021. With a median biologically effective dose of 71.7 Gy, a total dose of 56 Gy in 20 fractions was the most frequently used regimen (53.7%). The median age and Karnofsky Performance Status of patients were 74 years and 70, respectively. Most patients (*n* = 31, 77.5%) were diagnosed with glioblastoma, IDH-wildtype, CNS WHO grade 4. Only 10 (25.0%) patients underwent surgical resection, and 28 (70.0%) patients received concurrent temozolomide during hypoRT. With a median follow-up of 9.7 months, the median overall survival (OS) was 12.2 months. Of the 30 (75.0%) patients with disease progression, only 12 patients received salvage treatment. The OS after progression differed significantly depending on salvage treatment (median OS, 9.6 vs. 4.6 months, *p* = 0.032). Dose-escalated hypoRT in 20 fractions produced survival outcomes outperforming historical data for frail patients.

## 1. Introduction

According to the World Health Organization (WHO) classification for brain tumors, high-grade glioma (HGG) refers to WHO grade 3 or 4 tumors, which include, for example, glioblastoma, isocitrate dehydrogenase (IDH)-wildtype; astrocytoma, IDH-mutant; diffuse midline glioma, H3K27-altered tumors [1]. Typically, patients of the general population with HGG are subjected to surgical resection, followed by a total dose of 60 Gy in 30 fractions of radiotherapy (RT) with or without chemotherapy [2]. Nevertheless, those conventional therapeutic approaches have traditionally excluded elderly and frail patients due to the limited survival outcomes [3,4]. For frail patients with HGG who are old or have poor performance status, therefore, the National Comprehensive Cancer Network Guideline recommends RT with or without temozolomide, temozolomide alone, or best supportive care (BSC) as treatment options [2]. Based on a growing body of literature, the use of RT instead of BSC is supported for this patient group [5,6,7]. To minimize prolonged treatment periods, the efficacy of hypofractionated RT (hypoRT) with or without chemotherapy has been evaluated in multiple prospective trials [8,9,10,11,12]. HypoRT led to comparable but slightly inferior survival outcomes to those achieved with conventional fractionated RT (60 Gy). Based on these results, hypoRT according to several regimens (40–45 Gy in 15 fractions, 34 Gy in 10 fractions, or 25 Gy in 5 fractions) has been recommended for this patient cohort in current guidelines [2,13,14]. However, the biologically effective dose (BED, with α/β set to 10) of the aforementioned hypoRT regimens is lower than that of conventional fractionation with 60 Gy (BED 72 Gy vs. 38–42 Gy). Although dose-escalation trials showed no benefit over 60 Gy in HGG, comparable dose fractionation to 60 Gy might improve outcomes in frail patients [15]. Based on this assumption, we adopted a dose-escalated hypoRT regimen with 20 fractions, having a comparable BED to 60 Gy in 30 fractions (BED 72 Gy). In this retrospective study, we evaluated the clinical outcomes after a dose-escalated hypoRT regimen in frail patients with HGG.

## 2. Materials and Methods

### 2.1. Study Population

We retrospectively reviewed the medical records of frail patients diagnosed with radiologically or histologically confirmed HGG and treated with hypoRT at our institution between 2013 and 2021. Based on our institutional policy, hypoRT is considered for (1) patients aged  ≥70 (*n* = 27, 67.5%) and (2) patients aged <70 but with Karnofsky Performance Status (KPS) of  ≤70 (*n* = 13, 32.5%). A total of 40 patients were included in the final analysis. There was no patient who did not complete scheduled hypoRT. This study was approved by the Institutional Review Board (No. 2023-07-174), and the protocol adhered to the ethical guidelines of the 1975 Declaration of Helsinki. The requirement for informed consent was waived due to the retrospective nature of this study. The medical records and data were assessed for research purposes on 30 September 2023.

### 2.2. Treatment

The treatment strategy was determined through discussions with a multidisciplinary neuro-oncology board comprising neurosurgeons, radiologists, radiation oncologists, and medical oncologists. When surgery was performed, the extent of resection (EOR) was classified into four groups based on gadolinium-enhanced magnetic resonance imaging (MRI) performed within 48 h after surgery: gross total resection (GTR), with no visible contrast-enhanced residual tumor portion; subtotal resection, defined as the removal of ≥90% of the tumor volume; partial resection, defined as the removal of <90% of the tumor volume; and biopsy, defined as the performance of stereotactic biopsy only.

For patients with glioblastoma, IDH-wild-type, concurrent temozolomide (75 mg/m^2^ body surface area/day, 7 days/week, from the first to the last day of RT) was prescribed during RT, except for five patients who declined temozolomide. Conversely, patients with alternative tumor profiles received hypoRT as a standalone treatment.

For hypoRT, all patients underwent computed tomography (CT) simulations in the supine position, with a slice thickness of 2.5 mm. The gross tumor volume (GTV) and clinical target volume (CTV) were defined based on planning CT/MRI or diagnostic MRI. In particular, GTV1 included the surgical cavity, identified contrast-enhanced lesions, and suspected tumor-infiltrating regions with high signal intensity on T2 fluid-attenuated inversion recovery MRI sequences. GTV2 included all identified contrast-enhanced lesions. CTV1 was delineated by adding 1–2 cm margins to GTV1 to cover microscopic disease, while CTV2 and CTV3 were delineated by adding 0.5 and 0.3 cm margins to GTV2, respectively. All CTVs were modified according to the anatomic boundaries. The median GTV1 and CTV1 were 43.8 cc (interquartile range [IQR], 22.5–60.8) and 170.8 cc (IQR, 124.9–252.3), respectively. The planning target volume (PTV) included a CTV with a 3 mm margin for setup uncertainties.

The simultaneous integrated boost (SIB) technique was employed for 28 (70.0%) patients. The most used SIB regimen (*n* = 24, 60.0%) delivered total doses of 56 and 48 Gy in 20 fractions (BED 71.7 Gy and 59.5 Gy) to PTV2 and PTV1, respectively. Four patients (9.8%) received total doses of 64, 56, and 48 Gy in 20 fractions delivered to PTV3, PTV2, and PTV1 (BED 84.5 Gy, 71.7 Gy, and 59.5 Gy), respectively. For a single-dose prescription (*n* = 12, 30.0%), various dose regimens were used for PTV1: a total dose of 50 Gy in 20 fractions (BED 62.5 Gy, *n* = 4, 10.0%), 56 Gy in 20 fractions (BED 71.7 Gy, *n* = 5, 12.5%), 60 Gy in 20 fractions (BED 78.0 Gy, *n* = 2, 5.0%), or 44 Gy in 20 fractions (BED 53.7 Gy, *n* = 1, 2.5%). The median BED using an α/β of 10 (BED) was 71.7 Gy (IQR, 71.7–78.0). Three-dimensional conformal RT and intensity-modulated RT were utilized in four (10.0%) and 36 (90.0%) patients, respectively.

### 2.3. Endpoints and Statistical Analysis

The endpoints were overall survival (OS) and progression-free survival (PFS). For OS and PFS, the endpoints were calculated from the date of diagnosis to the date of the last follow-up or event. The OS of patients with recurrent disease was additionally calculated from the date of disease progression to the date of the last follow-up or death. The response was evaluated using radiological imaging according to the Response Assessment in Neuro-Oncology Working Group [16]. The first site of disease progression was classified as follows: local, defined as failure at the primary site or surgical cavity or within a 2 cm margin; noncontiguous intracranial, defined as intracranial failure beyond the range of local failure; and leptomeningeal seeding [17,18]. Survival outcomes were analyzed using the Kaplan–Meier method and compared using the log-rank test. The Cox proportional hazards regression model was used to estimate the hazard ratio (HR) and its 95% confidence interval (CI). Statistical significance was set at *p* < 0.05. Statistical analyses were performed using R software (version 4.2.3; R Foundation for Statistical Computing, Vienna, Austria).

## 3. Results

### 3.1. Baseline Characteristics

The baseline and treatment characteristics of patients are summarized in Table 1. Given the median age of 74 years (IQR, 68–81 years), we categorized patients according to whether their age was > 80 years. Twelve (30.0%) patients were > 80 years old, and 13 (32.5%) patients had a KPS < 70 (median KPS, 70 (IQR, 60–80)). More than half of patients (*n* = 30, 75.0%) underwent stereotactic biopsy. Among the 10 (25.0%) patients who underwent surgical resection, 3 (7.5%) patients received GTR. Most patients (*n* = 31, 77.5%) were diagnosed with glioblastoma, IDH-wild-type, CNS WHO grade 4. Temozolomide-based concurrent chemoradiotherapy (CCRT) was administered to 28 (70.0%) patients. 

### 3.2. Clinical Outcomes

**Figure 1:**
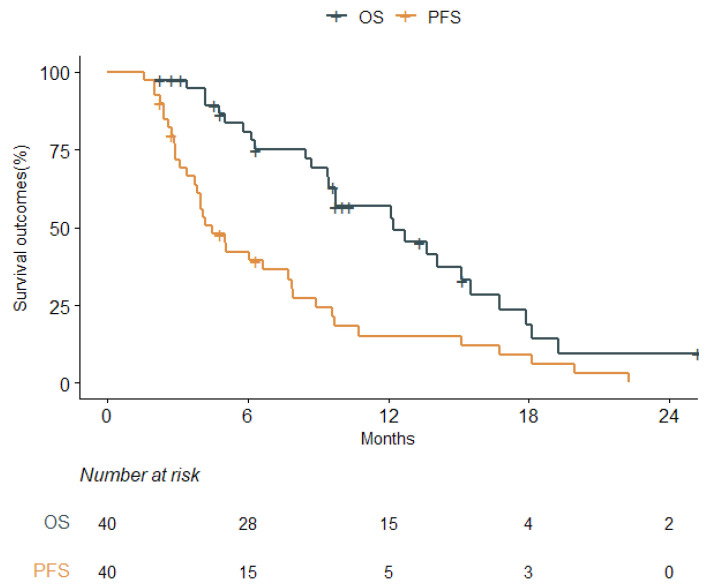
Kaplan–Meier survival curves of overall survival (OS) and progression-free survival (PFS) (*n* = 40).

During a median follow-up of 9.7 months, 30 patients (75.0%) experienced disease progression. The median OS and PFS of the entire cohort were 12.2 (95% confidence interval [CI], 9.7–16.8) and 4.5 (95% CI, 3.8–7.9) months, respectively. The 1-year OS and PFS were 57.0% and 15.2%, respectively (Figure 1). Neither EOR nor CCRT affected the OS or PFS outcomes. Also, BED ≥ 72 Gy and GTV were not associated with the OS or PFS outcomes. Overall, there were no statistically significant prognostic factors related to inferior OS or PFS outcomes (Table 2). In addition, there were no treatment-related adverse events of grade 3 or higher. Symptomatic pseudoprogression occurred in 15 patients (37.5%) following hypoRT. 

### 3.3. Salvage Treatment after Disease Progression

Among the 30 patients who experienced disease progression, 26 (86.6%), 2 (6.7%), and 2 (6.7%) had local failures, noncontiguous intracranial failures without local failure, and leptomeningeal seeding, respectively. Two of the 26 patients with local failure had simultaneous noncontiguous intracranial failures. 

**Figure 2:**
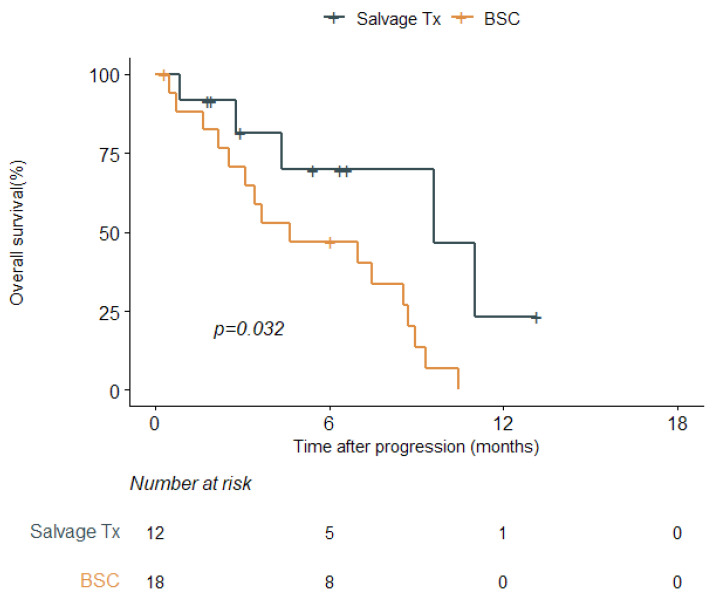
Kaplan–Meier survival curves for overall survival according to salvage treatment after disease progression (*n* = 30). Abbreviations: Tx, treatment; BSC, best supportive care.

After disease progression, 12 patients (40.0%) received salvage treatment (Table 3). Details of the salvage treatment are described in Table S1. Although no significant difference was observed in the patient or tumor characteristics according to salvage treatment, patients in the salvage-treatment group had a better performance status at the time of disease progression than those in the BSC group (KPS ≥ 70, 58.3% vs. 16.7% *p* = 0.048) (Table 3). After disease progression, salvage treatment was associated with better OS outcomes than BSC (median OS after disease progression, 9.6 [95% CI, 9.6–not reached] vs. 4.6 months [95% CI, 3.1–9.0], *p* = 0.032, Figure 2). Additionally, salvage treatment was the only significant prognostic factor for OS after disease progression (Table 4). Regarding salvage treatments, no significant difference was found between local and systemic therapy (median OS, 11.0 [95% CI, 2.8–not reached] vs. 9.6 months [95% CI, 4.4–not reached], *p* = 0.624, Figure S1).

## 4. Discussion

In this retrospective study, we analyzed the outcomes of dose-escalated hypoRT in 20 fractions to frail patients with HGG. Notably, salvage treatment rather than BSC was found to significantly improve survival after disease progression in this patient group. 

Following a trial by Keime-Guibert et al., which demonstrated a 3-month survival benefit of RT over BSC, multiple studies explored the optimal RT regimen for this patient cohort [6]. The efficacy of hypoRT in elderly patients with HGG has been extensively investigated (Table 5) [8,9,10,11,12,19,20]. Regarding fractionation, hypoRT with 40 Gy in 15 fractions (median OS of 5.6 months) or 34 Gy in 10 fractions (median OS of 7.5 months) resulted in comparable OS outcomes to the conventional 60 Gy in 30 fractions [10,11]. Regarding CCRT, prospective trials have revealed favorable survival outcomes in elderly patients [9,10]. Minniti et al. reported a median OS of 12.4 months in 71 patients aged ≥ 70 years treated with temozolomide-based concurrent hypoRT with 40 Gy in 15 fractions [9]. A randomized phase III trial by Perry et al. revealed an OS benefit after temozolomide-based concurrent hypoRT with 40 Gy in 15 fractions compared to hypoRT alone (median OS, 9.3 vs. 7.6 months, *p* < 0.001) [10]. At present, the optimal RT schemes for frail patients with HGG remain controversial. Current treatment guidelines recommend various hypoRT regimens (40 Gy in 15 fractions, 34 Gy in 10 fractions, or 25 Gy in 5 fractions) for this patient cohort to shorten the treatment period [2,13,14]. However, the BED of the aforementioned schemes is significantly lower than that of the conventional fractionation of 60 Gy in 30 fractions adopted for the general population (72 Gy vs. 37.5–50.7 Gy, Table 5). 

Several studies have revealed the OS benefit of conventional fractionation with 60 Gy in 30 fractions compared to hypoRT in elderly patients. Based on the National Cancer Database, Mak et al. revealed a survival benefit after conventional fractionation (58–63 Gy) compared with hypoRT (34–42 Gy), with and without chemotherapy [21]. In a recent large-scale Korean multi-institutional retrospective study by Wee et al., conventional fractionation of 60 Gy significantly improved OS from 13.3 months to 16.4 months compared to hypoRT with 45 Gy in 134 patients aged ≥ 70 years (*p* = 0.003) [20]. Despite the heterogeneous characteristics of the current cohort, hypoRT comprising 20 fractions (median BED, 71.7 Gy) resulted in a favorable median OS of 12.2 months even in older patients, compared to the results reported by Wee et al. The BED, which is similar to that of the conventional fractionation of 60 Gy, might lead to favorable outcomes. Although no evidence is available to support dose escalation over 60 Gy in 30 fractions for HGG, adopting a dose scheme comparable to 60 Gy may improve outcomes in frail patients with HGG [15]. Recently, 60 Gy in 20 fractions (BED 78 Gy) as an accelerated regimen resulted in a favorable median OS of 26.5 months in 89 non-frail patients [22]. Further investigations are required to identify the optimal RT dose scheme for this cohort. 

To date, only limited evidence is available regarding the optimal treatment for recurrent HGG in frail patients. Given the baseline physical function and quality of life of frail patients, treatment with the aim of salvage after recurrence is even more difficult to achieve. However, salvage treatment was associated with longer median OS than BSC (Figure 2 and Table 4). Zanello et al. reported that age ≥ 70 did not affect survival outcomes after recurrence [23]. They also found that any salvage treatment, including surgical resection, bevacizumab, and other systemic treatments, was associated with an OS benefit. Therefore, they suggested salvage treatment instead of BSC for elderly patients (≥70 years). Socha et al. also demonstrated that any salvage treatment, either local or systemic, could significantly improve post-progression survival compared to BSC (median, 23 vs. 9 weeks, *p* < 0.001) [24]. These researchers also identified a beneficial impact of salvage treatment over BSC on OS in patients with a poor KPS of < 60 at recurrence (median post-progression survival, 21 vs. 9 weeks, *p* = 0.014). In addition, a second surgery for recurrent tumors has been suggested to be effective and safe for this patient group [25]. Based on their study including 39 patients aged > 65 years, Farina Nunez et al. found that patients who underwent a second surgery after recurrence showed better OS outcomes than those that did not undergo a second surgery (median OS 18.0 vs. 10.1 months, *p* = 0.043) [25]. Although no difference in survival outcomes was found between local and systemic salvage treatments in the current cohort (Figure S1), an appropriate treatment option instead of BSC should be considered based on the performance status and extent of the disease in frail patients [26].

The current study had several limitations. As this was a single-center retrospective study, unrecognized biases may have existed that could not be fully addressed in the current analysis. Additionally, the small sample size may have resulted in an overestimation of the potential efficacy of hypoRT and salvage treatments for recurrent disease. While no severe treatment-related toxicities were observed in the study, it is important to conduct a more comprehensive analysis of the patients’ quality of life to support the safety of hypoRT. Moreover, the interpretation of current survival outcomes should consider the heterogeneity of histologic diagnoses. However, we conducted an initial exploration of the potential efficacy of dose-escalated hypoRT comprising 20 fractions for frail patients with mostly nonresected tumors (75.0%). We found a favorable OS compared to historical data. 

In conclusion, this hypoRT regimen with a median BED of 71.7 Gy in 20 fractions could provide relief from complaints due to the longer treatment duration of 30 fractions, while still delivering a higher BED than historical hypoRT regimens (40–45 Gy in 15 fractions, 34 Gy in 10 fractions, or 25 Gy in 5 fractions) and a similar BED to the standard regimen of 60 Gy in 30 fractions. Furthermore, salvage treatment after recurrence resulted in improved survival outcomes compared with BSC, even in frail patients. Further investigations are needed to explore the efficacy and safety of dose-escalated hypoRT regimens in frail patients with HGG.

## Figures and Tables

**Table 1:** Patient, tumor, and treatment characteristics.

Characteristics	N (%)
**Age, years**	
Median [IQR]	74 [68–81]
≤80	28 (70.0)
>80	12 (30.0)
**Sex**	
Female	15 (37.5)
Male	25 (62.5)
**KPS**	
Median [IQR]	70 [60–80]
<70	13 (32.5%)
≥70	27 (67.5%)
**Extent of resection**	
Biopsy	30 (75.0)
Partial resection	1 (2.5)
Subtotal resection	6 (15.0)
Gross total resection	3 (7.5)
**Pathology**	
Glioblastoma, IDH-wildtype	31 (77.5)
Diffuse midline glioma H3K27M-mutant	3 (7.5)
Astrocytoma, IDH-wildtype	4 (10.0)
Astrocytoma, IDH-mutant	2 (5.0)
**MGMT promoter methylation**	11 (27.5)
**Temozolomide-based CCRT**	28 (70.0)
**Adjuvant temozolomide**	25 (62.5)

Values are expressed as number of patients (%) or median [interquartile range]. IQR, interquartile range; KPS, Karnofsky Performance Status; IDH, isocitrate dehydrogenase; WHO, World Health Organization; CNS, central nervous system; MGMT, O(6)-methylguanine DNA methyltransferase; and CCRT, concurrent chemoradiotherapy.

**Table 2:** Outcomes and prognostic factors for overall survival and progression-free survival.

Overall Survival						
Variables		Event ^a^	Median (Months)	HR	95% CI	*p*-Value
Age, years	≤80	19/28	12.2	Reference	-	0.799
	>80	8/12	14.1	0.89	0.38–2.11	
Sex	Female	8/15	15.1	Reference	-	0.158
	Male	19/25	9.1	1.83	0.79–4.24	
KPS	<70	9/13	9.5	Reference	-	0.689
	≥70	18/27	12.2	1.19	0.51–2.78	
Extent of resection	GTR	1/3	8.7	Reference	-	
	STR/PR	6/7	9.5	1.15	0.13–10.2	0.903
	bx/no bx	20/30	12.7	1.23	0.16–9.47	0.842
MGMT promoter	Methylated	6/11	13.6	Reference	-	0.873
	Others	21/29	12.1	1.08	0.43–2.69	
CCRT	No	8/12	13.6	Reference	-	0.368
	Yes	19/28	12.2	1.50	0.62–3.61	
Adjuvant TMZ	No	8/15	12.1	Reference	-	0.840
	Yes	19/25	12.7	0.92	0.40–2.12	
BED, Gy	<72	20/29	12.2	Reference	-	0.871
	≥72	7/11	15.5	0.93	0.39–2.24	
GTV, cc	Continuous	43.8 ^b^	12.2	1.00	1.00–1.01	0.114
**Progression-Free Survival**						
**Variables**		**Event ^a^**	**Median (Months)**	**HR**	**95% CI**	***p*** **-Value**
Age, years	≤80	25/28	4.5	Reference	-	0.613
	>80	11/12	5.8	0.83	0.39–1.73	
Sex	Female	15/15	4.1	Reference	-	0.899
	Male	21/25	5.0	1.05	0.53–2.07	
KPS	<70	11/13	6.7	Reference	-	0.127
	≥70	25/27	4.5	1.82	0.84–3.94	
Extent of resection	GTR	2/3	7.9	Reference	-	
	STR/PR	7/7	5.1	1.03	0.20–5.24	0.971
	bx/no bx	27/30	4.2	1.33	0.31–5.66	0.702
MGMT promoter	Methylated	8/11	5.0	Reference	-	0.935
	Others	28/29	4.2	1.03	0.46–2.30	
CCRT	No	10/12	4.2	Reference	-	0.247
	Yes	26/28	4.5	1.61	0.72–3.59	
Adjuvant TMZ	No	13/15	3.1	Reference	-	0.103
	Yes	23/25	6.7	0.55	0.27–1.13	
BED, Gy	<72	27/29	4.5	Reference	-	0.825
	≥72	9/11	4.6	0.92	0.43–1.97	
GTV, cc	Continuous	43.8 ^b^	4.5	1.00	1.00–1.01	0.579

HR, hazard ratio; CI, confidence interval; KPS, Karnofsky Performance Status; GTR, gross total resection; STR, subtotal resection; PR, partial resection; bx, biopsy; MGMT, O(6)-methylguanine-DNA methyltransferase; CCRT, concurrent chemoradiotherapy; TMZ, temozolomide; BED, biologically effective dose; and GTV, gross tumor volume. For the BED calculation, an α/β ratio of 10 was used. ^a^ The value is presented as number of events/number of patients in the group. ^b^ Values are presented as medians.

**Table 3:** Patient characteristics according to salvage treatment after disease progression.

Characteristics	Total *N* = 30	Salvage Tx *N* = 12	BSC *N* = 18	*p*-Value
**Initial treatment**				
**Age, years** (median, [IQR])	74 [67–81]	78 [66–81]	72 [67–81]	0.496
**Sex**				0.501
Female	14 (46.7)	7 (58.3)	7 (38.9)	
Male	16 (53.3)	5 (41.7)	11 (61.1)	
**KPS**				1.000
<70	9 (30.0)	4 (33.3)	5 (27.8)	
≥70	21 (70.0)	8 (66.7)	13 (72.2)	
**Extent of resection**				0.199
Biopsy/no surgery	22 (73.3)	8 (66.7)	14 (77.8)	
Partial/subtotal resection	6 (20.0)	2 (16.7)	4 (22.2)	
Gross total resection	2 (6.7)	2 (16.7)	0 (0.0)	
**Pathology**				0.273
Glioblastoma, IDH-wild-type	22 (73.3)	7 (58.3)	15 (83.3)	
Others	8 (26.7)	5 (41.7)	3 (16.7)	
**MGMT promoter methylation**	6 (20.0)	2 (16.7)	4 (22.2)	1.000
**Temozolomide-based CCRT**	21 (70.0)	7 (58.3)	14 (77.8)	0.464
**Adjuvant temozolomide**	19 (63.3)	6 (50.0)	13 (72.2)	0.395
**RT modality**				1.000
3D CRT	3 (10.0)	1 (8.3)	2 (11.1)	
IMRT	27 (90.0)	11 (91.7)	16 (88.9)	
**Total dose, Gy** (median, [IQR])	56 [56–59]	56 [56–58]	56 [56–59]	0.631
**BED, Gy** (median, [IQR])	71.7 [71.7–76.4]	71.7 [71.7–74.9]	71.7 [71.7–76.4]	0.631
**GTV, cc** (median, [IQR])	46.5 [22.9–61.9]	46.5 [21.8–60.5]	44.8 [23.0–64.4]	0.692
**CTV, cc** (median, [IQR])	176.5 [121.9–289.8]	153.1 [104.6–207.0]	230.3 [143.0–318.6]	0.079
**At disease progression**				
**Recurrence-free interval**	4.0 [2.8–7.9]	7.2 [3.0–8.8]	3.8 [2.8–5.1]	0.189
**Age, years** (median, [IQR])	74 [67–81]	78 [67–82]	72 [67–81]	0.432
**KPS**				0.048
<70	20 (66.7%)	5 (41.7%)	15 (83.3%)	
≥70	10 (33.3%)	7 (58.3%)	3 (16.7%)	
**Site of disease progression**				0.478
Local	26 (86.6%)	11 (91.7%)	15 (83.3%)	
Noncontiguous intracranial failure	2 (6.7%)	1 (8.3%)	1 (5.6%)	
Leptomeningeal seeding	2 (6.7%)	0 (0.0%)	2 (11.1%)	

Values are expressed as number of patients (%) or median [interquartile range]. Abbreviations: Tx, treatment; BSC, best supportive care. IQR, interquartile range; KPS, Karnofsky Performance Status; IDH, isocitrate dehydrogenase; MGMT, O(6)-methylguanine-DNA methyltransferase; CCRT, concurrent chemoradiotherapy; RT, radiotherapy; 3D CRT, three-dimensional conformal RT; IMRT, intensity-modulated RT; BED, biologically effective dose; GTV, gross tumor volume; and CTV, clinical target volume. For BED calculation, an α/β ratio of 10 was used.

**Table 4:** Prognostic factors for overall survival after disease progression.

Variables		Event ^a^	Median (Months)	HR	95% CI	*p*-Value
Age, years	≤80	14/20	7.5	Reference	-	0.159
	>80	7/10	4.4	2.02	0.76–5.36	
Sex	Female	7/14	7.0	Reference	-	0.536
	Male	14/16	7.5	1.35	0.53–3.44	
KPS at	<70	15/20	7.5	Reference	-	0.141
progression	≥70	6/10	8.3	0.46	0.16–1.30	
Initial EOR	GTR	1/2	0.8	Reference	-	
	STR/PR	5/6	3.4	0.87	0.10–7.83	0.903
	Bx/no Bx	15/22	7.5	0.62	0.08–4.88	0.648
MGMT	Methylated	4/6	8.9	Reference	-	0.432
promoter	Others	17/24	7.0.	1.56	0.52–4.71	
Management	BSC	16/18	4.6	Reference	-	0.032
at progression	Salvage Tx	5/12	9.6	0.29	0.10–0.90	
RFI, month	Continuous	4.0 ^b^	7.0	0.99	0.91–1.08	0.891

HR, hazard ratio; CI, confidence interval; KPS, Karnofsky Performance Status; EOR, extent of resection; GTR, gross total resection; STR, subtotal resection; PR, partial resection; Bx, biopsy; MGMT, O(6)-methylguanine-DNA methyltransferase; Tx, treatment; BSC, best supportive care; and RFI, recurrence-free interval. ^a^ The value is presented as number of events/number of patients in the group. ^b^ Values are presented as medians.

**Table 5:** Studies assessing radiotherapy for frail patients with high-grade glioma.

Study	Inclusion Criteria	No.	CCRT (%)	Surgery (%)	RT Regimen	BED (EQD_2_) (Gy)	Median OS
**Roa [11]**	≥60 y and KPS ≥ 50	48	0	65	40 Gy/15 fx	50.7 (42.2)	5.6 m
**Malmstrom [8]**	60–70 y and ECOG PS 0–2	58	0	78	34 Gy/10 fx	45.6 (38.0)	8.8 m
	>70 y and ECOG PS 0–2	40	0	67	34 Gy/10 fx	45.6 (38.0)	7.0 m
**Wick [19]**	>65 y and KPS ≥ 60	178	0	61	60 Gy/30 fx	72.0 (60.0)	9.4 m
**Minniti [9]**	≥70 y and KPS ≥ 60	71	100	87	40 Gy/15 fx	50.7 (42.2)	12.4 m
**Perry [10]**	>65 y and ECOG PS 0–2	281	100	68	40 Gy/15 fx	50.7 (42.2)	9.3 m
		281	0	68	40 Gy/15 fx	50.7 (42.2)	7.6 m
**Wee [20]**	>65 y	196	100	88	60 Gy/30 fx	72.0 (60.6)	17.6 m
		64	100	67	45 Gy/15 fx	58.5 (48.8)	13.2 m
**Current** **study**	>70 y or KPS ≤ 70	28	100	29	56 Gy/20 fx	71.7 (59.7)	12.2 m
		12	0	17	56 Gy/20 fx	71.7 (59.7)	13.6 m

CCRT, concurrent chemoradiotherapy; RT, radiation therapy; BED, biologically effective dose; EQD_2_, equivalent doses at 2 Gy per fraction; OS, overall survival; KPS, Karnofsky Performance Status; ECOG PS, Eastern Cooperative Oncology Group performance status; fx, fractions; ‘m’ and ‘y’ represent ‘months’ and ‘years’, respectively. For the BED and EQD_2_ calculations, an α/β ratio o f10 was used.

## Data Availability

The datasets generated and analyzed during the current study are available from the corresponding author on reasonable request.

## Supplementary Materials

The following supporting information can be downloaded at: https://www.mdpi.com/article/10.3390/cancers16010064/s1, Figure S1: Kaplan–Meier survival curves for overall survival according to management after disease progression (*n* = 30); Table S1: Details of treatment after disease progression (*n* = 30).
