# Supplementary material for: Treatment Outcomes after Dose-Escalated Moderately Hypofractionated Radiotherapy for Frail Patients with High-Grade Glioma
Source: Cancers (Basel). 2023 Dec 21;16(1):64. doi: 10.3390/cancers16010064 (PMC10778244; doi:10.3390/cancers16010064)
Supplement: Supplementary file 1 [file cancers-16-00064-s001.zip › cancers-2748234-supplementary.pdf]

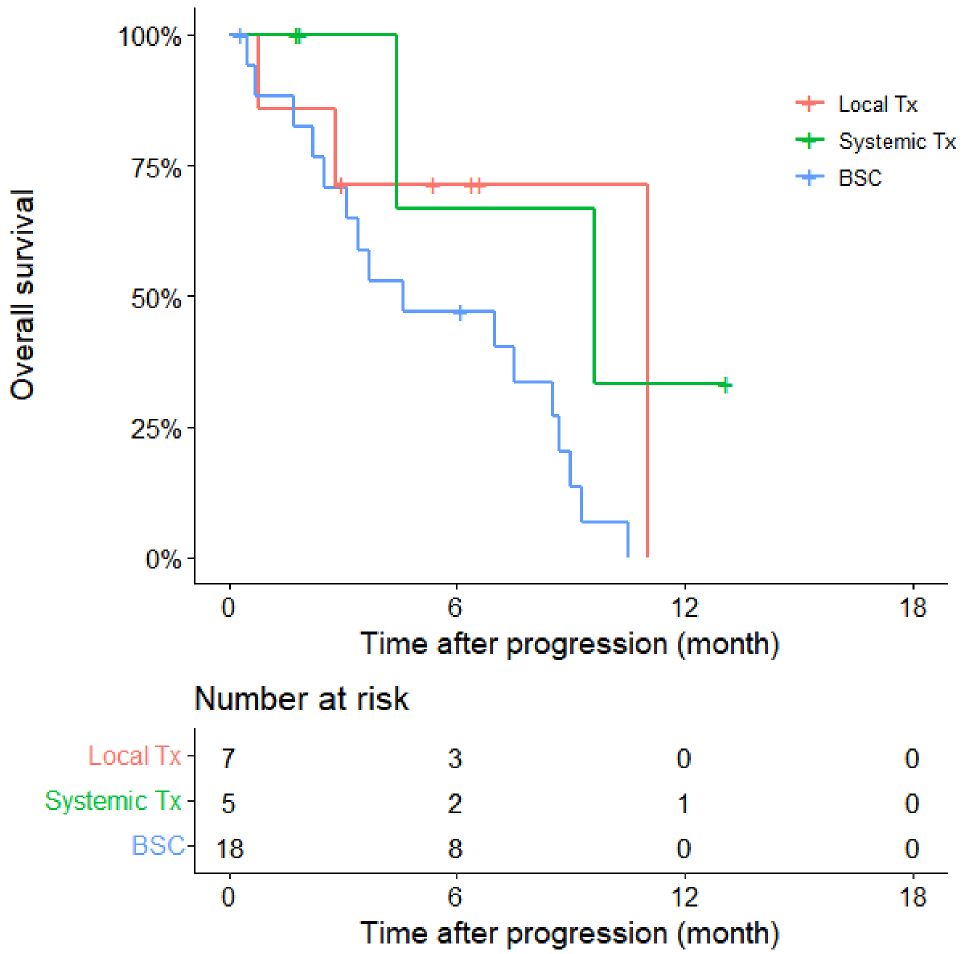

Figure S1. Fig. Kaplan–Meier survival curves for overall survival according to management after disease progression (n = 30). Abbreviations: Tx, treatment; BSC, best supportive care.

Table S1. Details of treatment after disease progression (n = 30).

No.	Salvage Tx	Details	Further PD	RFI to 2nd PD (month)
**1**	Local	2nd surgery	yes	4.83
**2**	Local	GKS	yes	1.37
**3**	Local	GKS		
**4**	Local	GKS, Bevacizumab/Irinotecan#1 → Bevacizumab #6		
**5**	Local	GKS + Bevacizumab #7	yes	4.63
**6**	Local	GKS, low dose-TMZ #1		
**7**	Local	GKS, low dose TMZ #2		
8	Systemic	Bevacizumab /Irinotecan #2		
**9**	Systemic	Bevacizumab/Irinotecan #1		
**10**	Systemic	PCV #1		
**11**	Systemic	TMZ #3	yes	2.2
**12**	Systemic	TMZ #2	yes	1.9
**13-30**	BSC			

Abbreviations: Tx, treatment; PD, progressive disease; RFI, Recurrence free interval; GKS, gamma knife surgery; TMZ, temozolomide; PCV, procarbazine, lomustine and vincristine; BSC, best supportive care. BSC means management to control any disease related symptoms including steroid, mannitol, anti-epileptic drug, etc.
